# Rare Variants in Genes of the Cholesterol Pathway Are Present in 60% of Patients with Acute Myocardial Infarction

**DOI:** 10.3390/ijms232416127

**Published:** 2022-12-17

**Authors:** Ricardo Pan-Lizcano, Luis Mariñas-Pardo, Lucía Núñez, Fernando Rebollal-Leal, Domingo López-Vázquez, Ana Pereira, Aranzazu Molina-Nieto, Ramón Calviño, Jose Manuel Vázquez-Rodríguez, Manuel Hermida-Prieto

**Affiliations:** 1 Grupo de Investigación en Cardiología, Instituto de Investigación Biomédica de A Coruña (INIBIC), Complexo Hospitalario Universitario de A Coruña (CHUAC-SERGAS), GRINCAR-Universidade da Coruña (UDC), 15006 A Coruña, Spain; 2 Facultad de Ciencias de la Salud, Universidad Internacional de Valencia (VIU), 46002 Valencia, Spain; 3 Departamento de Ciencias de la Salud, GRINCAR Research Group, Universidade da Coruña, 15403 A Coruña, Spain; 4 Servicio de Cardiología, Complexo Hospitalario Universitario de A Coruña (CHUAC-SERGAS), Instituto de Investigación Biomédica de A Coruña (INIBIC), Universidade da Coruña (UDC), 15006 A Coruña, Spain; 5 CIBERCV (Centro de Investigación Biomédica en Red Enfermedades Cardiovasculares), Instituto de Salud Carlos III, 28029 Madrid, Spain

**Keywords:** acute myocardial infarction, cholesterol genes, rare variants

## Abstract

Acute myocardial infarction (AMI) is a pandemic in which conventional risk factors are inadequate to detect who is at risk early in the asymptomatic stage. Although gene variants in genes related to cholesterol, which may increase the risk of AMI, have been identified, no studies have systematically screened the genes involved in this pathway. In this study, we included 105 patients diagnosed with AMI with an elevation of the ST segment (STEMI) and treated with primary percutaneous coronary intervention (PPCI). Using next-generation sequencing, we examined the presence of rare variants in 40 genes proposed to be involved in lipid metabolism and we found that 60% of AMI patients had a rare variant in the genes involved in the cholesterol pathway. Our data show the importance of considering the wide scope of the cholesterol pathway in order to assess the genetic risk related to AMI.

## 1. Introduction

Acute myocardial infarction (AMI) is defined as myocardial cell death due to prolonged ischemia [1]. It is the most severe type of coronary artery disease (CAD) and one of the main causes of death in developed countries [2].

Epidemiological studies have identified the following three major categories of risk factors for AMI: unchangeable factors (age, gender, and family history); variable factors (smoking, alcohol intake, lack of exercise, poor diet, high blood pressure, diabetes, dyslipidemia, and metabolism syndrome); and emerging factors (abnormal levels of C-reactive protein (CRP), fibrinogen, coronary artery calcification (CAC), homocysteine, and lipoprotein(a)) [3]. The environmental and lifestyle components of this triad of AMI have been well-documented since the 1960s, starting with the Framingham study [4]. However, it Is important to note that genetic predisposition has been stated to account for 40–50% of the variability in the development of CAD [4,5].

However, to date, the range of genes underlying the heritable component of AMI is not fully known. According to Musunuru et al. (2016) [6], 25% of AMI loci are lipid-related, including the following: *LDLR*; *PCSK9*; *APOB*; *SORT1*; *ABCG5/G8*; *LPA*; *ABO*; *TCF21*; *SH2B3*; *APOE*; *APOA1/A5*; *LPL*; *TRIB1*; and *LIPA*. These data reinforce the role of cholesterol homeostasis in the genetic landscape of AMI. According to the Kyoto Encyclopedia of Genes and Genomes (KEGG), the main cholesterol pathway includes more than 40 genes that operate on many planes, including cholesterol uptake, efflux, transport, storage, utilization, and/or excretion [7].

Most studies [8,9,10,11,12,13,14,15,16] have focused on the analysis of a limited number of genes in the cholesterol pathway, converging on the secretion of the HDL and LDL molecules, without considering the complexity of the pathway as a whole. Most of these studies have focused on single nucleotide polymorphisms (SNPs) [12,14,15,16,17] without taking into account the study of rare variants in the whole pathway. An ambitious strategy of studying the rare variants in the whole pathway is important as a rare variant can be considered, not the cause of the disease, but an additional risk factor [18,19,20].

Thus, in this paper we aimed to study potential rare variants in the cholesterol pathway genes in patients who presented AMI in order to obtain a broader spectrum of variants that may be involved.

## 2. Results

### 2.1. Characteristics of the Study Participants

The principal characteristics of the 105 patients included in the study are summarized in Table 1. The data are also shown to be disaggregated between patients with mutation and without mutation. There was not a significant difference between the groups in any of these variables.

### 2.2. Classification of Variants Identified in the Cholesterol Pathway

**Figure 1:**
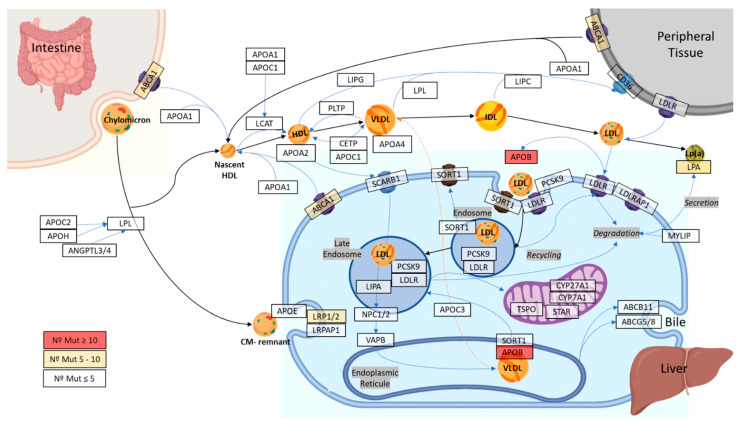
Schematic view of the 40 selected genes in the cholesterol metabolism pathway.

The 40 genes of the cholesterol metabolism pathway (Figure 1) presented a mean coverage of 117.06 ± 22.14-fold (Figure S1).

After the analysis of the sequencing data, 474 unique genetic variants in the codifying region, and ±10 intronic bases of the 40 genes related to cholesterol metabolism, were identified. Considering the variants in the 105 patients, the total number of variants was 8805. On average, each patient carried around 83 variants in the analyzed region.

**Figure 2:**
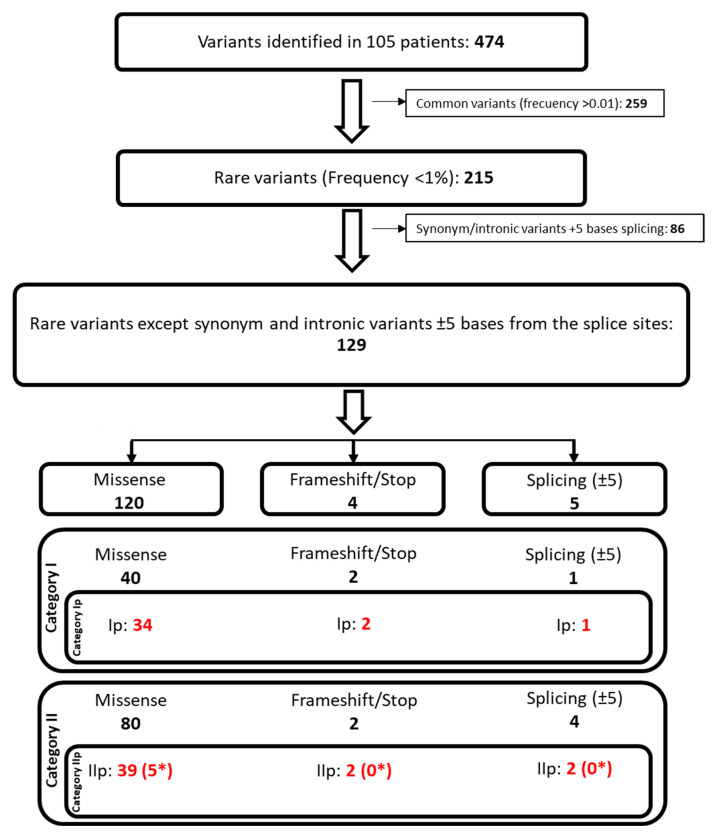
Schematic view of the followed steps for the filtering and classification of the variants identified in the patients of study. * Variants non-previously described. Red: category Ip (known mutation) and IIp (potential mutation) variants.

Next, the filtering strategy described in the methods section was applied (Figure 2) in order to discriminate between the relevance of each variant. First, 259 common variants, with a frequency of higher than 0.01 in the population, were filtered out, leaving 215 rare variants. Second, the synonymous and intronic variants in more than ±5 bases on the splicing sites were excluded, reducing the variants to 129 (86 filtered out). Those 129 variants were classified using Hass et al.’s classification [21], with slight modifications which are presented in the methods section.

From the 129 selected variants, 43 had already been described in the Human Gene Mutation Database (HGMD) [22], classified following our strategy as category I (Table 2, [23,24,25,26,27,28,29,30,31,32,33,34,35,36,37,38,39,40,41,42,43,44,45,46,47,48,49,50,51,52,53,54,55,56,57,58,59,60,61,62,63,64,65,66,67,68,69,70,71,72,73,74,75,76,77]). A total of 37 of the 43 variants were related to a lipid metabolism disorder or to cardiopathy. Consequently, they were classified as category Ip (known mutation). The mutations categorized as Ip were present in 18 genes, including the top three genes with the most mutations in our patients, namely *APOB* (*n* = 11), *ABCA1* (*n* = 4), and *LDLR* (*n* = 3) (Table 2).

The remaining 86 variants were classified as category II. After the assessment of their potential impact on the protein by in silico prediction tools, 46 variants were classified as potentially damaging and, consequently, were classified as category IIp (potential mutation) (Table 3). In this group, the genes *APOB* (*n* = 7), *LRP1* (*n* = 6), and *LRP2* (*n* = 5) were the most represented.

Five new missense variants (IIp) were described, namely *ABCA1*-p.A2058G, *ABCG5*-p.P431A, *APOB*-p.D2193Y, *APOB*-p.A4119D, and *PCSK9*-p.A300V (Table 3). As for all IIp variants, at least three out of the five in silico tools predicted an impact on protein function (Mutation taster, SNAP2, SIFT, PP2, PhD-SNP).

### 2.3. Distribution of the Variants of Interest in the Genes

**Figure 3:**
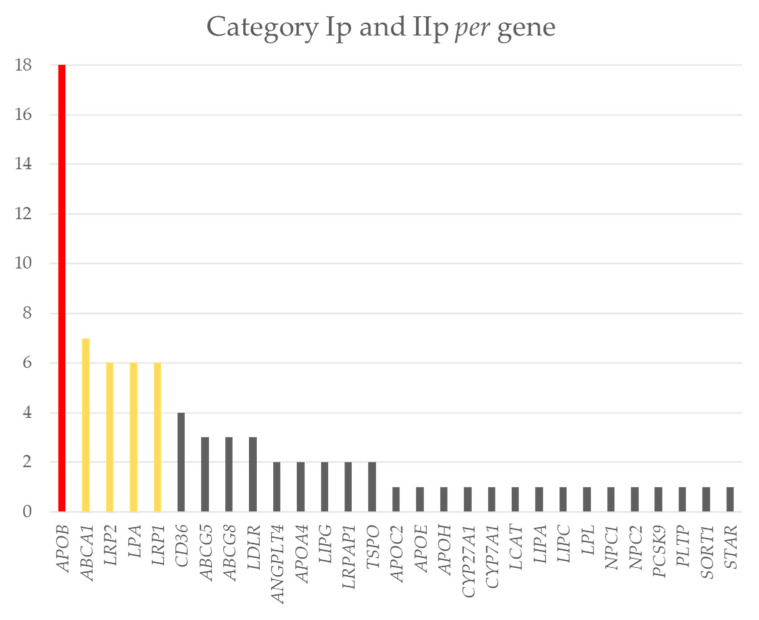
Bar graph displaying the number of mutations and potential mutations (Y axis) *per* gene (X axis). Red: more than 8 variants; yellow: between 8–6 variants; grey: less than 6 variants.

A total of 80 mutations and potential mutations (category Ip and IIp) were identified in the studied population. These variants were present in 72% of the genes studied (29 out of 40) (Figure 3). The distribution of variants in the genes was uneven. *APOB* was the gene in which more of these variants were identified (*n* = 18). Then, genes *LRP2*, *ABCA1*, *LPA*, and *LRP1* had between six to eight variants. Finally, the 24 remaining genes had between one to three variants of category Ip (known mutation) and IIp (potential mutation) mutations (Figure 3). These group had 44% of the variants identified in our population.

### 2.4. Distribution of the Variants of Interest in the Patients

**Figure 4:**
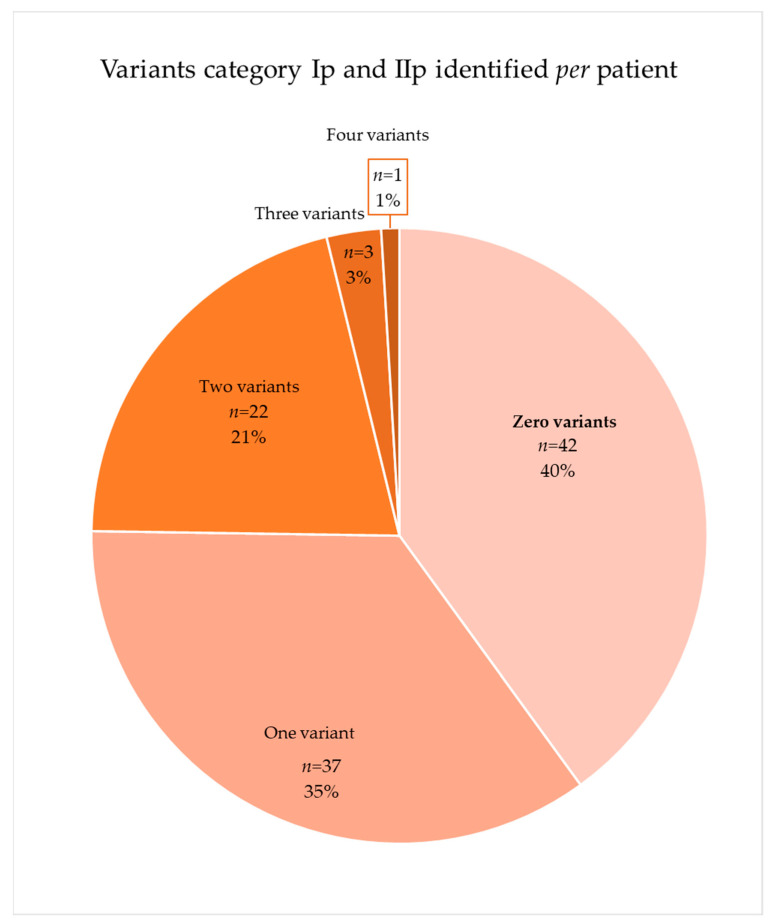
Distribution of patients with category Ip (known mutation) and IIp (potential mutation).

The distribution of the 80 variants included in category Ip (known mutation) and IIp (potential mutation) showed that approximately 60% of the patients (*n* = 63) had a mutation or potential mutation in one of the genes analyzed (Figure 4). The 37 variants of category Ip (known mutation) appeared 50 times in 43 patients, and 7 of these patients had more than one variant. The 43 variants of category IIp (potential mutation) appeared 44 times in 37 patients of which 7 had at least two variants. 

## 3. Discussion

In the present study, we examined the presence of rare variants in 40 genes proposed to be involved in lipid metabolism in AMI patients, and we identified mutations in the genes of this pathway in 60% of patients. Therefore, our data highlight the importance of analyzing rare variants in patients with AMI in the cholesterol pathway.

The importance of the cholesterol pathway has been stressed in GWAS studies that show the association between loci in lipid-related genes and susceptibility to AMI [78,79,80,81,82,83,84,85]. The association between SNPs in genes, such as APOB [85], LDLR [19,86,87], PCSCK9 [88], and AMI has been described, while other studies have reported a weak association between variants in ABCA1 and both the incidence of AMI and the risk of symptomatic CAD [89]. 

Although common sequence variants have been extensively studied in large genome-wide association studies, it is also important to understand the contribution of rare variants to the susceptibility of AMI [18,90]. For this purpose, the selection of the genes implicated in the cholesterol pathway and the strategy used to identify rare variants associated with AMI were two key points of this study.

One of the genes studied the most in order to identify low-frequency lipid-associated variants with AMI is LDLR, which codifies the low-density lipoprotein receptor. Approximately 5% of patients with CAD and AMI under the age of 60 years carry heterozygous LDLR mutations [19,86,87]. These mutations are present and equally distributed throughout the gene in the form of exonic substitutions, small exonic rearrangements, large rearrangements, promoter variants, intronic variants, variants in the 3′ untranslated sequence, point mutations, splice site mutations, and large deletions [87]. In our study, two missense variants, p.G269D and p.G592E, were identified in one patient each while a third variant, p.T726I, was identified in two patients. These data imply a frequency of variants in the LDLR gene of 0.038, similar to those described in the literature. These three variants were previously described in patients with AMI [91]. However, the impact on the protein is not fully understood because in one study, the authors were not able to observe a disruptive effect on LDL uptake [91]. Meanwhile, in other research [92], it was demonstrated that patients who carried the mutation presented levels of 50% for LDLR expression, LDL-LDLR binding, and LDLR uptake.

However, in our study, the major gene in which variants were found was APOB, which codified the primary apolipoprotein, including 100 chylomicrons, as well as VLDL, Lp(a), IDL, and LDL particles [93]. In fact, 18 variants were identified in the *APOB* gene in 19 patients, which implies a frequency of mutations in this gene in our cohort of 0.181. It is important to note that one patient carried three variants of the *APOB* gene (p.P145S, p.T741N, p.L1212M) and that the variants p.T3826M and p.R1128H were present in two and three patients, respectively. Of the 18 variants found in the *APOB* gene, 11 were previously described as being mainly associated with hypercholesterolemia and hypertriglyceridemia (category Ip (known mutation)), while 7 were only listed in databases, such as Gnomad, with a frequency of less than 0.01 (Category IIp (potential mutation)). Surprisingly, 9 out of 11 of the variants in category Ip (known mutation) were not previously associated with AMI [37,39,43,94,95,96,97,98]. However, two of them, namely p.P2821L and p.S2429T, have been described in patients with cardiovascular artery diseases [39,45,46]. Moreover, our study is in concordance with previous studies that suggest that rare deleterious mutations in *APOB*, such as R3500Q/W, confer a higher risk of ischemic cardiovascular disease in mutation carriers [99]. In fact, the importance of APOB levels has been stressed because the Copenhagen City Heart Study showed that apolipoprotein B levels are associated with ischemic diseases [99].

The most important finding of this study was that 60% of AMI patients had a rare variant in genes involved in the cholesterol pathway. This fact confirms the importance of not focusing only on the gene with a higher presence of rare variants, such as LDLR in the literature or *APOB* in our case, but rather on the 40 selected genes of the cholesterol pathway. In our study, we have found more than three mutations in *LRP2*, *ABCA1* [89], *LPA*, *LRP1*, *CD36* genes. Additionally, we found between one and three rare variants in genes such as LDLR and PCSK9 which, in other studies, had a high impact on their association with AMI [78,79,88,100]. In fact, the low frequency of variants in the *LDLR* and *PCSK9* genes in the group is surprising and supports the idea of the importance of analyzing rare variants in the entire pathway of cholesterol genes.

It is important to highlight that 11 genes of the 40 included, in which we did not identify rare variants, have been described in the literature to have an association with AMI [101,102,103,104,105,106,107,108,109,110,111]. Therefore, these genes should be further analyzed in following studies in order to increase the 60% of patients with rare variants detected in this study.

The main limitation of this study is that it relies on the fact that it was a descriptive study, not an association study. The sample size is small for an association study of rare variants that present a frequency lower than 0.01. We have not been able to associate the variants with cholesterol levels because in many cases there was no previous data on cholesterol levels prior to infarction due to AMI being the first event. Moreover, because a functional validation of each variant was not performed, this method may have led to a misclassification in some cases.

AMI is a pandemic in which the risk of patients can be assessed; however, conventional risk factors are inadequate to detect who is at risk early in the asymptomatic stage [4]. Genetic risks, which can be determined at birth, could help to identify patients at higher risk of presenting AMI [4]. Our data show that rare variants in genes related to cholesterol are present in AMI patients. In future clinical settings where genomic sequencing might be available for all patients, the evaluation of genetic risks would be improved by incorporating variants of the genes of the cholesterol pathway.

## 4. Materials and Methods

### 4.1. Study Population

In this study, 105 patients diagnosed with AMI with an elevation of the ST segment (STEMI) and treated with primary percutaneous coronary intervention (PPCI) in A Coruña University Hospital (Spain) between July 2017 to January 2021, were included.

Written informed consent was obtained from every patient included in the study. The protocol of this study was in accordance with the principles of the Declaration of Helsinki, and the “Comité de Ética de la Investigación de Galicia” (ref: 2016/299) also approved it. Blood samples were collected at the time of the hemodynamic procedure and stored at −80 °C until analysis. All samples were included in the biobank of the National Biobank Network of “Instituto de Salud Carlos III” (C.0002483, 2013/109).

A chi-squared test was performed for the following variables: sex; dyslipidemia; hypertension; diabetes; and tobacco, as these variables were considered dichotomous. For age, an ANOVA test was performed.

### 4.2. Selection of Genes Related to Cholesterol Metabolism

To establish the main genes involved in the cholesterol metabolism pathway, the KEGG map04979 and WikiPathways WP4522 databases were analyzed. Therefore, 40 genes related to the cholesterol metabolism pathway were selected. All genes selected are involved in cholesterol uptake, efflux, transport, storage, utilization, and/or excretion.

### 4.3. Next-Generation Sequencing

Targeted resequencing was performed using two different kits: the TruSight One sequencing kit of Illumina (San Diego, California, USA) (*n* = 22) and the Exome Research Panel v.2 of IDT (Leuven, Belgium) (*n* = 83). The sequencing reactions were conducted on a NextSeq500 platform of Illumina (San Diego, California, USA). The TruSight One kit yielded the sequencing of approximately 5000 genes and the Exome Research Panel v.2 yielded the sequencing of around 20,000 genes. Both strategies included the selected 40 genes related to cholesterol metabolism (Table S1).

The strategy for exome sequence data analysis included several computational tools. The raw data files in the binary base call (BCL) format, generated by the NextSeq Sequencing System, were demultiplexed and converted to a standard FASTQ file by bcl2fastq Conversion Software v2.17. The coverage (Figure S1) and Q-score were analyzed in order to establish the quality of the sequence. Based on the guidelines of the American College of Medical Genetics and Genomics, if the region analyzed presented a sequencing mean depth <30, the region was considered unsuitable for analysis. Furthermore, the threshold in the Q-score established was 30 (base call accuracy of 99.9%). 

Computational biology sequence alignment to the human genome version GRCh37/hg19 was performed with Burrows–Wheeler Aligner software 7.17, and the BAM file output was obtained. Variants were detected using the Genome Analysis Toolkit (GATK), and the output files were VCF files. All exonic regions and ±10 intronic regions of the 40 genes related to cholesterol metabolism were visualized via the Integrative Genome Viewer (IGV).

### 4.4. Variant Annotation and Classification

All variants identified in this study were searched in the Genome Aggregation Database (gnomAD) [112] and the Single Nucleotide Polymorphism Database (dbSNP) [113] in order to establish frequency in the general population. Moreover, to identify previous genotype–phenotype associations, all variants were checked in HGMD [22].

After the annotation of all variants, variant filtering was performed using an adapted variant filtering and prioritization strategy described by Akinrinade et al. [114] (Figure 2). In detail, the first filter used was the frequency of the variants was described in the Gnomad and/or dbSNP to be higher than 0.01. This frequency was selected following the classical classification of common variants. The second filtering excluded the synonymous and intronic variants located farther than five bases from the acceptor/donor splice sites.

The remaining variants were classified into well-defined categories described by Hass et al. [21] with slight modifications. We defined two categories with a subcategory each for the determination of the likelihood of being disease-relevant mutations (Figure 2): (a) Category I consisted of variants previously described in the literature (HGMD) to be associated with pathologies. Variants of this group associated with lipid metabolism disorders or cardiopathies were classified as Ip (known mutation). (b) Category II included all variants that were not previously pathology-associated in the literature. The subcategory IIp (potential mutation) included variants with more than three out of five in silico predictions tools to be pathogenic and all the stop-gained variants.

The in silico prediction tools used differed between the types of variants. The potential effect of the missense variants was predicted using five different tools: Mutation Taster (http://www.mutationtaster.org (accessed on 28 October 2022)) [115]; SNAP2 (https://www.rostlab.org/services/snap/ (accessed on 28 October 2022)) [116], SIFT (http://sift.jcvi.org/www/SIFT_seq_submit2.html (accessed on 28 October 2022)) [117]; Polyphen2 (http://genetics.bwh.harvard.edu/%20pph2/ (accessed on 28 October 2022)) [118]; and PhD-SNP (http://snps.uib.es/phd-snp/phdsnp.html (accessed on 28 October 2022)) [119]. For the synonym and intronic variants located ± 5 bases from the acceptor/donor splice sites, the potential effect was assessed with five different tools: Mutation Taster (http://www.mutationtaster.org (accessed on 28 October 2022)) [115]; Splice Site Prediction by Neural Network of Berkeley Drosophila Genome Project (https://www.fruitfly.org/seq_tools/splice.html (accessed on 28 October 2022)) [120]; NetGene2–2.42 (https://services.healthtech.dtu.dk/service.php?NetGene2-2.42 (accessed on 28 October 2022)) [121]; Alternative Splice Site Predictor (http://wangcomputing.com/assp/index.html (accessed on 28 October 2022)) [122]; and CADD-Splice (https://cadd.gs.washington.edu/snv (accessed on 28 October 2022)) [123]. As suggested by Houdayer et al. [124], it is considered a potential pathogenic variant if the variation of the score given by the prediction tool, between the wildtype and the variant, is at 20% or is more significant. 

Thus, category Ip (known mutation) mutations were considered known mutations and category IIp (potential mutation) mutations were considered potential mutations.

## 5. Conclusions

A high prevalence of rare variants in the genes of the cholesterol pathway can be found in AMI patients. Our data show the need to consider not only one gene or a small set of genes, but the wide scope of cholesterol pathway genes in order to make a more realistic assessment of the risks related to AMI.

## Figures and Tables

**Table 1:** Clinical data and demographic characteristics of the studied population.

		All Patients (*n* = 105)	With Mutations (*n* = 63)	Without Mutations (*n* = 42)
**Age (years)**		57.89 ± 12.12	55.35 ± 11.61	59.29 ± 12.31
**Sex (M, %)**		80	74.6	88.1
**Dyslipidemia (%)**		50.50	46.30	57.14
**Treatment of dyslipidemia (%)**				
**Hypertension (%)**		46.66	42.85	52.38
**Diabetes (%)**		17.15	17.46	16.66
**Tobacco (%)**		69.52	74.60	61.90
**AMI Localization (%)**	**Anterior**	56	60,30	50
	**Septal**	2	1.50	2.40
	**Inferior**	35	30.20	42.80
	**Posterior**	1	1.50	0
	**Lateral**	4	5	2.40
	**Indeterminate**	2	1.50	2.40
**Vessels affected (%)**	**1**	51	47.60	57.20
	**2**	22	22.20	21.40
	**3**	27	30.20	21.40
**Time of Ischemia (%)**	**<120 min**	17	13	21.40
	**120–360 min**	76	81	71.50
	**>360 min**	7	6	7.10

All data are referred to in terms of percentage, except age.

**Table 2:** Category Ip (known mutation).

Gene	Position	dbSNP Code	c.HGVS	Type	p.HGVS	References	Described Pathology in HGMD
*ABCA1*	9:107558416	rs528270977	c.5300A>G	Missense	p.Y1767C	[23]	Reduced total cholesterol
	9:107589238	rs138880920	c.2328G>C	Missense	p.K776N	[24,25,26]	Increased risk of ischemic heart disease
	9:107599376	rs9282543	c.1196T>C	Missense	p.V399A	[27,28,29]	Tangier disease
	9:107646756	rs145183203	c.254C>T	Missense	p.P85L	[29,30,31]	HDL deficiency
*ABCG5*	2:44065739	rs56204478	c.80G>C	Missense	p.G27A	[32,33]	Hypercholesterolaemia
*ABCG8*	2:44101610	rs370422066	c.1476T>A	Stop gained	p.Y492*	[34]	Phytosterolaemia
	2:44102301	rs761153163	c.1505C>T	Missense	p.P502L	[35]	Sitosterolaemia
*ANGPTL4*	19:8436373	rs140744493	c.1006C>T	Missense	p.R336C	[28,36,37]	Lower plasma triglyceride level
*APOA4*	11:116691720	rs147577451	c.1054A>T	Missense	p.N352Y	[34]	High triglyceride
	11:116692293	rs12721043	c.481G>T	Missense	p.A161S	[32,37,38]	Hyperlipidaemia
*APOB*	2:21225354	rs72654423	c.12940A>G	Missense	p.I4314V	[39]	Hypercholesterolaemia
	2:21228263	rs61744153	c.11477C>T	Missense	p.T3826M	[32,40,41]	Hypertriglyceridaemia
	2:21228339	rs12713540	c.11401T>A	Missense	p.S3801T	[40]	Hypercholesterolaemia
	2:21230828	rs72653098	c.8912A>C	Missense	p.N2971T	[42,43]	Familial hypercholesterolemia
	2:21231278	rs72653095	c.8462C>T	Missense	p.P2821L	[44,45]	Hypocholesterolaemia
	2:21232455	rs72653092	c.7285T>A	Missense	p.S2429T	[46,47,48]	Hypertriglyceridaemia
	2:21234674	rs151009667	c.5066G>A	Missense	p.R1689H	[46,49]	Hypertriglyceridaemia
	2:21238367	rs12713843	c.3383G>A	Missense	p.R1128H	[29,50,51]	Hypobetalipoproteinaemia
	2:21238413	rs12713844	c.3337G>C	Missense	p.D1113H	[37,51,52]	Hypobetalipoproteinaemia
	2:21249682	rs12714192	c.2222C>A	Missense	p.T741N	[37]	Dyslipidaemia
	2:21260934	rs6752026	c.433C>T	Missense	p.P145S	[37]	Dyslipidaemia
*APOC2*	19:45452024	rs120074114	c.122A>C	Missense	p.K41T	[29,37,53]	Apolipoprotein C2 deficiency
*APOE*	19:45411110	rs769452	c.137T>C	Missense	p.L46P	[48]	Hypercholesterolaemia
*APOH*	17:64210599	rs150652035	c.973T>G	Missense	p.C325G	[54,55,56]	Apolipoprotein H deficiency
*CD36*	7:80292426	rs138897347	c.550G>A	Missense	p.D184N	[57]	CD36 deficiency
*CYP27A1*	2:219679730	rs374507635	c.1573C>T	Stop gained	p.Q525*	[58]	Cerebrotendinous xanthomatosis
*LDLR*	19:11217352	rs143992984	c.806G>A	Missense	p.G269D	[48,59,60]	Hypercholesterolaemia
	19:11227604	rs137929307	c.1775G>A	Missense	p.G592E	[48,61,62]	Hypercholesterolaemia
	19:11233886	rs45508991	c.2177C>T	Missense	p.T726I	[63,64,65]	Hypercholesterolaemia
*LIPA*	10:90988005	rs544080483	c.380G>A	Missense	p.R127Q	[66]	Hypercholesterolaemia
*LIPG*	18:47109939	rs138438163	c.1171G>A	Missense	p.E391K	[34,67,68]	Higher plasma HDL cholesterol
	18:47109955	rs77960347	c.1187A>G	Missense	p.N396S	[34,69,70]	Higher plasma HDL cholesterol
*LPA*	6:160966559	rs139145675	c.5311C>T	Missense	p.R1771C	[71]	Plasminogen deficiency
	6:160969693	rs143431368	c.4974-2A>G	Splice acceptor	-	[31,72]	Lowered human lipoprotein(a) levels
*LRP2*	2:170042245	rs35734447	c.9613A>G	Missense	p.N3205D	[73]	Hypoplastic left heart syndrome
*NPC2*	14:74953134	rs151220873	c.88G>A	Missense	p.V30M	[74,75,76]	Niemann-Pick disease, type C2
*SORT1*	1:109910100	rs61797119	c.370A>G	Missense	p.I124V	[32,77]	Hypercholesterolaemia

cHGVS: change in the gene-coding sequence; pHGVS: amino acid change in the protein.

**Table 3:** Category IIp (potential mutation).

Gene	Position	dbSNP Code	cHGVS	Type	pHGVS	*In Silico* Prediction Programs								
						M.T.	SNAP2	SIFT	PP2	PhD-SNP	BDGP	NetGene2	ASSP	C. Splice
*ABCA1*	9:107549242	rs1230573600	c.6220G>A	Missense	p.G2074S	PP (0.999)	PP (69)	PP (0.00)	PP (0.999)	PP (7)				
	9:107550232	9:107550232	c.6173C>G	Missense	p.A2058G	PP (0.999)	PP (31)	PP (0.00)	PP (0.998)	PP (6)				
	9:107599296	rs201586430	c.1276T>C	Missense	p.F426L	PP (0.999)	PP (50)	PP (0.00)	PP (0.999)	PP (4)				
*ABCG5*	2:44040359	rs137996263	c.1852T>C	Missense	p.S618P	PP (0.998)	PP (51)	PP (0.00)	PP (0.945)	PP (8)				
	2:44051085	2:44051085	c.1291C>G	Missense	p.P431A	PP (0.999)	PP (9)	NPP (0.94)	PP (1)	NPP (4)				
*ANGPTL4*	19:8435981	rs866158597	c.703G>A	Missense	p.V235M	PP (0.999)	PP (28)	NPP (0.14)	PP (0.999)	PP (7)				
*APOB*	2:21225938	2:21225938	c.12356C>A	Missense	p.A4119D	NPP (0.999)	PP (56)	PP (0.00)	PP (0.989)	PP (1)				
	2:21227295	rs1458765902	c.11933T>C	Missense	p.I3978T	PP (0.999)	PP (63)	PP (0.00)	PP (0.977)	PP (0)				
	2:21229970	rs146178619	c.9770A>G	Missense	p.N3257S	PP (0.984)	PP (4)	PP (0.00)	NPP (0.073)	PP (5)				
	2:21230600	rs61742323	c.9140C>T	Missense	p.T3047M	NPP (0.999)	PP (17)	PP (0.00)	PP (0.625)	NPP (6)				
	2:21233163	2:21233163	c.6577G>T	Missense	p.D2193Y	NPP (0.999)	PP (42)	PP (0.00)	NPP (0.396)	PP (0)				
	2:21238007	rs61736761	c.3634C>A	Missense	p.L1212M	NPP (0.989)	PP (10)	PP (0.00)	PP (1)	NPP (7)				
	2:21246505	rs773987185	c.2496G>A	Missense	p.M832I	PP (0.738)	NPP (-55)	PP (0.03)	PP (0.592)	NPP (4)				
*CD36*	7:80276161	rs754478799	c.107del	Frameshift variant	p.K36Rfs*41	PP (1)	-	-	-	-				
	7:80293767	rs201715989	c.655G>T	Missense	p.D219Y	PP (0.998)	PP (47)	PP (0.01)	NPP (0.139)	PP (9)				
	7:80299343	rs748146857	c.818+5G>A	Splicing variant	-	PP (1)					Diff. 24.24%	Diff. 34.04%	Diff. 11.06%	PP (21.3)
*CYP7A1*	*CYP7A1*/8:59405037	rs149291486	c.1090C>T	Missense	p.R364W	PP (0.999)	PP (93)	PP (0.00)	PP (1)	PP (9)				
*LCAT*	16:67976376	rs1186446170	c.638A>G	Missense	p.Y213C	PP (0.989)	PP (29)	PP (0.01)	PP (1)	PP (6)				
*LIPC*	15:58838165	rs540524619	c.799G>T	Missense	p.G267C	PP (0.999)	PP (60)	PP (0.01)	PP (1)	PP (8)				
*LPA*	6:160966559	rs139145675	c.5311C>T	Missense	p.R1771C	PP (0.999)	PP (20)	PP (0.00)	PP (1)	PP (7)				
	6:160969591	rs757921434	c.5074C>T	Stop gained	p.R1692*	PP (1)	-	-	-	-				
	6:160998167	rs200099994	c.4631+1G>A	Splicing variant	-	PP (1)					Diff. >20%	-	Diff. >20%	PP (31)
	6:161006084	rs76144756	c.4283C>T	Missense	p.P1428L	NPP (0.996)	PP (33)	PP (0.02)	PP (1)	PP (5)				
*LPL*	8:19819628	rs116403115	c.1325T>G	Missense,	p.V442G	PP (0.999)	PP (22)	PP (0.02)	PP (1)	NPP (3)				
*LRP1*	12:57549979	rs750499142	c.1330C>T	Missense	p.R444C	PP (0.999)	PP (44)	PP (0.00)	PP (1)	PP (8)				
	12:57577915	rs141826184	c.5977C>T	Missense	p.R1993W	PP (0.971)	PP (58)	PP (0.00)	PP (1)	PP (7)				
	12:57587039	rs113379328	c.7636G>A	Missense	p.G2546S	PP (0.996)	NPP (-19)	NPP (0.58)	PP (0.742)	PP (3)				
	12:57599365	rs149488896	c.11495G>C	Missense	p.G3832A	PP (0.997)	PP (26)	PP (0.04)	PP (0.999)	NPP (2)				
	12:57601936	rs755903131	c.11975G>A	Missense	p.R3992H	PP (0.999)	PP (6)	NPP (0.22)	PP (0.998)	PP (7)				
	12:57606021	rs142605462	c.13471G>C	Missense	p.D4491H	PP (0.999)	PP (25)	NPP (0.15)	PP (1)	NPP (4)				
*LRP2*	2:169997031	rs746070288	c.13133C>T	Missense	p.P4378L	PP (0.999)	PP (27)	NPP (0.17)	PP (1)	PP (1)				
	2:170034493	rs145432614	c.10213G>A	Missense	p.G3405R	PP (0.999)	PP (28)	NPP (0.48)	PP (0.907)	PP (4)				
	2:170037997	rs1248351989	c.10130A>C	Missense	p.D3377A	PP (0.999)	PP (38)	NPP (0.25)	PP (1)	PP (1)				
	2:170058335	rs750566206	c.8255G>A	Missense	p.R2752Q	PP (0.999)	PP (14)	PP (0.00)	PP (0.999)	PP (5)				
	2:170163815	rs142594441	c.403G>A	Missense	p.D135N	PP (0.999)	PP (2)	PP (0.00)	PP (1)	PP (7)				
*LRPAP1*	4:3519802	rs760183295	c.710G>A	Missense	p.R237H	PP (0.999)	PP (13)	-	PP (0.546)	NPP (3)				
	4:3521804	rs141393177	c.466C>T	Missense	p.H156Y	PP (0.996)	PP (10)	-	PP (0.934)	NPP (9)				
*NPC1*	18:21152041	rs762610198	c.284C>T	Missense	p.S95F	PP (0.999)	PP (23)	PP (0.01)	NPP (0.022)	PP (3)				
*PCSK9*	1:55521765	1:55521765	c.899C>T	Missense	p.A300V	PP (0.999)	PP (66)	PP (0.01)	PP (1)	PP (7)				
*PLTP*	20:44530943	rs6065903	c.1138C>T	Missense	p.R380W	NPP (0.551)	PP (68)	PP (0.00)	PP (1)	NPP (6)				
*STAR*	8:38005810	rs748942681	c.214G>A	Missense	p.E72K	PP (0.999)	PP (65)	PP (0.02)	NPP (0.358)	PP (1)				
*TSPO*	22:43557122	rs746919529	c.247G>C	Missense	p.G83R	PP (0.999)	PP (1)	NPP (0.28)	PP (1)	PP (4)				
	22:43557156	rs142445069	c.281C>T	Missense	p.A94V	PP (0.999)	PP (37)	NPP (0.36)	PP (0.805)	PP (5)				

M.T.: Mutation taster; PP2: PolyPhen2; BDGP: splice site predictor; ASSP: alternative splice site predictor; C. Splice: combined annotation-dependent depletion splice; Diff: difference; PP: predicted pathogenic; NPP: non-predicted pathogenic; cHGVS: change in the gene-coding sequence; pHGVS: amino acid change in the protein.

## Supplementary Materials

The following supporting information can be downloaded at: https://www.mdpi.com/article/10.3390/ijms232416127/s1.
