# Supplementary material for: Rare Variants in Genes of the Cholesterol Pathway Are Present in 60% of Patients with Acute Myocardial Infarction
Source: Int J Mol Sci. 2022 Dec 17;23(24):16127. doi: 10.3390/ijms232416127 (PMC9786046; doi:10.3390/ijms232416127)
Supplement: Supplementary file 1 [file ijms-23-16127-s001.zip › ijms-2044882-supplementary.pdf]

Supplementary materials: Table S1 and Figure S1

Table S1: Genes analyzed in the cholesterol pathway

KEGG Entry	Gene Symbol	Name	ENSEMBLE ID	Number of variants
K05641	*ABCA1*	ATP-binding cassette, subfamily A (ABC1), member 1	ENSG00000165029	31
K05664	*ABCB11*	ATP-binding cassette, subfamily B (MDR/TAP), member 11	ENSG00000073734	14
K05683	*ABCG5*	ATP-binding cassette, subfamily G (WHITE), member 5 (sterolin 1)	ENSG00000138075	9
K05684	*ABCG8*	ATP-binding cassette, subfamily G (WHITE), member 8 (sterolin 2)	ENSG00000143921	18
K22288	*ANGPTL3*	Angiopoietin-like 3	ENSG00000132855	3
K08767	*ANGPTL4*	Angiopoietin-like 4	ENSG00000167772	8
K08757	*APOA1*	Apolipoprotein A-I	ENSG00000118137	1
K08758	*APOA2*	Apolipoprotein A-II	ENSG00000158874	1
K08760	*APOA4*	Apolipoprotein A-IV	ENSG00000110244	9
K14462	*APOB*	Apolipoprotein B	ENSG00000084674	59
K22286	*APOC1*	Apolipoprotein C-I	ENSG00000130208	1
K22287	*APOC2*	Apolipoprotein C-II	ENSG00000234906	1
K08759	*APOC3*	Apolipoprotein C-III	ENSG00000110245	1
K04524	*APOE*	Apolipoprotein E	ENSG00000130203	6
K17305	*APOH*	Beta-2-glycoprotein 1	ENSG00000091583	12
K06259	*CD36*	CD36 antigen	ENSG00000135218	8
K16835	*CETP*	Cholesteryl ester transfer protein	ENSG00000087237	8
K00488	*CYP27A1*	Cholestanetriol 26-monooxygenase	ENSG00000135929	4
K00489	*CYP7A1*	Cholesterol 7alpha-monooxygenase	ENSG00000167910	1
K00650	*LCAT*	Lecithin-cholesterol acyltransferase	ENSG00000213398	3
K12473	*LDLR*	Low-density lipoprotein receptor	ENSG00000130164	17
K12474	*LDLRAP1*	Low density lipoprotein receptor adapter protein 1	ENSG00000157978	3
K01052	*LIPA*	Lysosomal acid lipase/cholesteryl ester hydrolase	ENSG00000107798	4
K22283	*LIPC*	Hepatic triacylglycerol lipase	ENSG00000166035	11
K22284	*LIPG*	Endothelial lipase	ENSG00000101670	5
K09644	*LPA*	Apolipoprotein(a)	ENSG00000198670	32
K01059	*LPL*	Lipoprotein lipase	ENSG00000175445	10
K04550	*LRP1*	Low-density lipoprotein receptor-related protein 1	ENSG00000123384	48
K06233	*LRP2*	Low-density lipoprotein-related protein 2	ENSG00000081479	61
K22290	*LRPAP1*	Alpha-2-macroglobulin receptor-associated protein	ENSG00000163956	15
K10637	*MYLIP*	E3 ubiquitin-protein ligase MYLIP	ENSG00000007944	5
K12385	*NPC1*	Niemann-Pick C1 protein	ENSG00000141458	18
K13443	*NPC2*	Niemann-Pick C2 protein	ENSG00000119655	1
K13050	*PCSK9*	Proprotein convertase subtilisin/kexin type 9	ENSG00000169174	18
K08761	*PLTP*	Phospholipid transfer protein	ENSG00000100979	2
K13885	*SCARB1*	Scavenger receptor class B, member 1	ENSG00000073060	7
K12388	*SORT1*	Sortilin	ENSG00000147465	7
K16931	*STAR*	Steroidogenic acute regulatory protein, mitochondrial	ENSG00000147465	4
K05770	*TSPO*	Translocator protein	ENSG00000100300	8
K10707	*VAPB*	ALS8 vesicle-associated membrane protein-associated protein B	ENSG00000124164	0

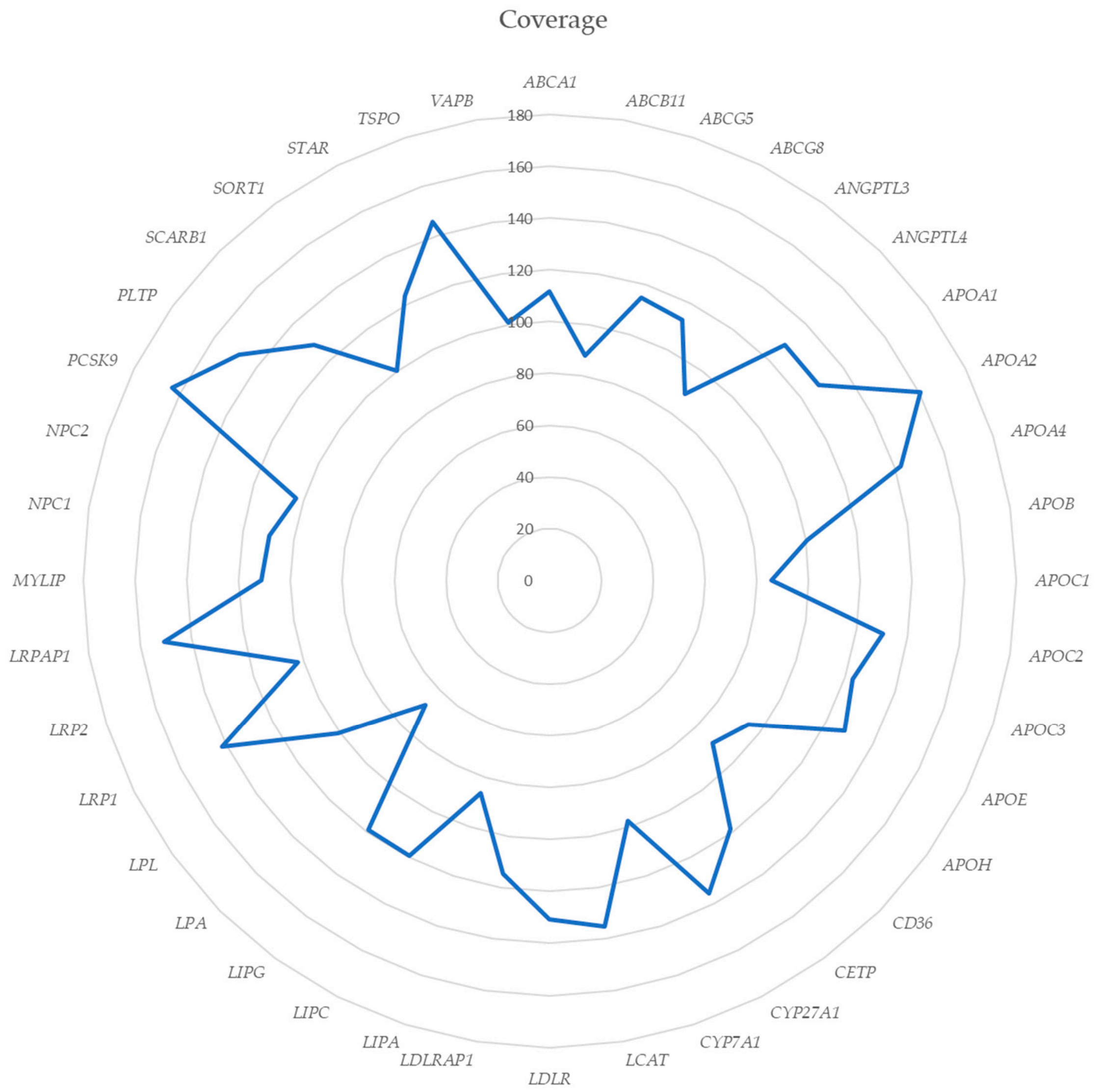

Figure S1: Mean gene coverage.
